# Minimising mutation load as a mechanism for low-dose hyper-radiosensitivity and induced radioresistance

**DOI:** 10.1038/s41598-026-65215-y

**Published:** 2026-08-25

**Authors:** Szabolcs Polgár, Balázs G. Madas

**Affiliations:** 1 Doctoral School of Physics, ELTE Eötvös Loránd University, Budapest, Hungary; 2 Environmental Physics Department, Institute for Energy Security and Environmental Safety, HUN-REN Centre for Energy Research, Budapest, Hungary

**Keywords:** Biological physics, Computational models, Systems biology

## Abstract

Low-dose hyper-radiosensitivity (HRS) and induced radioresistance (IRR) are unexpected features of cellular survival curves that challenge classical radiobiological models. While often interpreted phenomenologically, their underlying biological purpose remains unclear. Here we propose that these effects reflect an evolved strategy by which tissues minimise mutational burden through context-dependent cell elimination. We introduce the Minimum Mutation Load (MML) model, a principle-based framework in which irradiated cells assess their survival based on local intercellular signals that reflect neighbourhood damage. This cooperative behaviour balances the benefit of removing highly damaged cells with the mutational cost of their replacement. Using a curated dataset of 99 clonogenic survival experiments, we show that the MML model replicates key features of HRS and IRR across diverse conditions, with an average adjusted R² of 0.74, and performs comparably to the established Induced Repair (IR) model. Unlike the IR model, which is phenomenological, the MML model provides biologically interpretable parameters with independent theoretical grounding. The model suggests that mutation minimisation may be an organising principle of tissue homeostasis. These findings support a new conceptual framework in which tissue-level cooperation, rather than purely cell-intrinsic responses, governs somatic maintenance and cancer suppression.

## Introduction

Cancer can significantly reduce the lifespan of multicellular organisms. While natural selection does not strongly act against post-reproductive mortality in general, the reproductive success of organisms with high parental investment is also jeopardized by cancer risk. In these organisms, therefore, natural selection tends to favour biological mechanisms and processes that reduce cancer risk.

Derényi and Szöllősi^1^ argue that one of the main functions of tissue-specific stem cells and differentiation hierarchies is to reduce cancer risk. They demonstrate that a sufficiently large number of progressively slower-dividing cell types can limit the mutation load nearly as effectively as non-renewing tissues. Their model offers a quantitative framework for understanding how hierarchical tissue organization constrains unwanted somatic evolution, including the development of cancer.

Similarly, Rodriguez-Brenes et al.^2^ investigated how key features of cell lineages—including the number of intermediate cell compartments, self-renewal capacity, and division rates—affect the replication capacity of cells, which is a key determinant of cancer risk. They showed that a lower average replication capacity among dividing cells reduces the likelihood of bypassing replication limits during abnormal growth. Their findings highlight that the architecture of cell lineages plays a critical role in limiting the replicative potential of cells, and may have evolved as a cancer-protective strategy.

If one of the major reasons for the emergence of tissue hierarchies is the reduction of cancer risk, then it is reasonable to assume that additional protective mechanisms exist to actively eliminate cells with high levels of mutagenic damage. Within each level of the tissue hierarchy, such mechanisms could selectively favour the survival or proliferation of cells bearing fewer mutations. Any form of active selection against heavily damaged cells—whether through apoptosis, cell cycle arrest, or other processes—would be advantageous for the organism. However, since cell division itself is a major source of mutations, excessive cell death could inadvertently increase the overall mutation burden by necessitating additional rounds of proliferation.

In order to find evidence for such a selective mechanism, we focus on experiments using a well-known mutagenic agent, ionising radiation. Ionising radiation enables controlled induction of mutations in cells, making it a useful tool for investigating whether mutation-minimising selection occurs within a tissue among cells on the same hierarchical level. The clonogenic assay, or colony formation assay, is a fundamental technique of radiobiology used to study clonogenic survival after irradiation. It determines the ability of a single seeded cell to divide and form a colony, which indicates survival when 50 or more cells are counted. The technique was first introduced by Puck et al.^3^. Direct cell death and cell inactivation both result in the inability to form colonies; thus, in this study, we consider cell inactivation to be equivalent to cell death, in accordance with the principles of the clonogenic assay.

While clonogenic survival assays are in vitro and cannot fully replicate the complex architecture, signalling environment and cellular hierarchy of living tissues, they remain the gold standard for quantifying cellular radiosensitivity^4^. One major challenge in interpreting these data is the lack of tissue-level context, such as paracrine signalling gradients, immune-cell interactions, and tissue architecture. In vitro systems, however, may still retain critical intracellular and intercellular mechanisms^5–7^, including elements of damage recognition, repair pathways, and even coordinated cell fate decisions. Notably, the presence or absence of HRS and IRR varies significantly between cell lines, which may reflect the partial retention or loss of cooperative damage response mechanisms.

Using the clonogenic assay technique, a colony of cells can be examined by irradiating them together and then observing the survival of individual cells. In the classical clonogenic cell survival assay, the proportion of surviving cells usually decreases exponentially with increasing dose^8^. This exponential response is described by a linear and a quadratic component, hence the name Linear–Quadratic (LQ) model. The LQ model is expressed by the following equation:

1 $$\:SF={e}^{-\alpha\:D-\beta\:{D}^{2}}$$

where *D* is the applied dose, and *α* and *β* are parameters that characterise the radiosensitivity of the cells^9^.

**Fig. 1:**
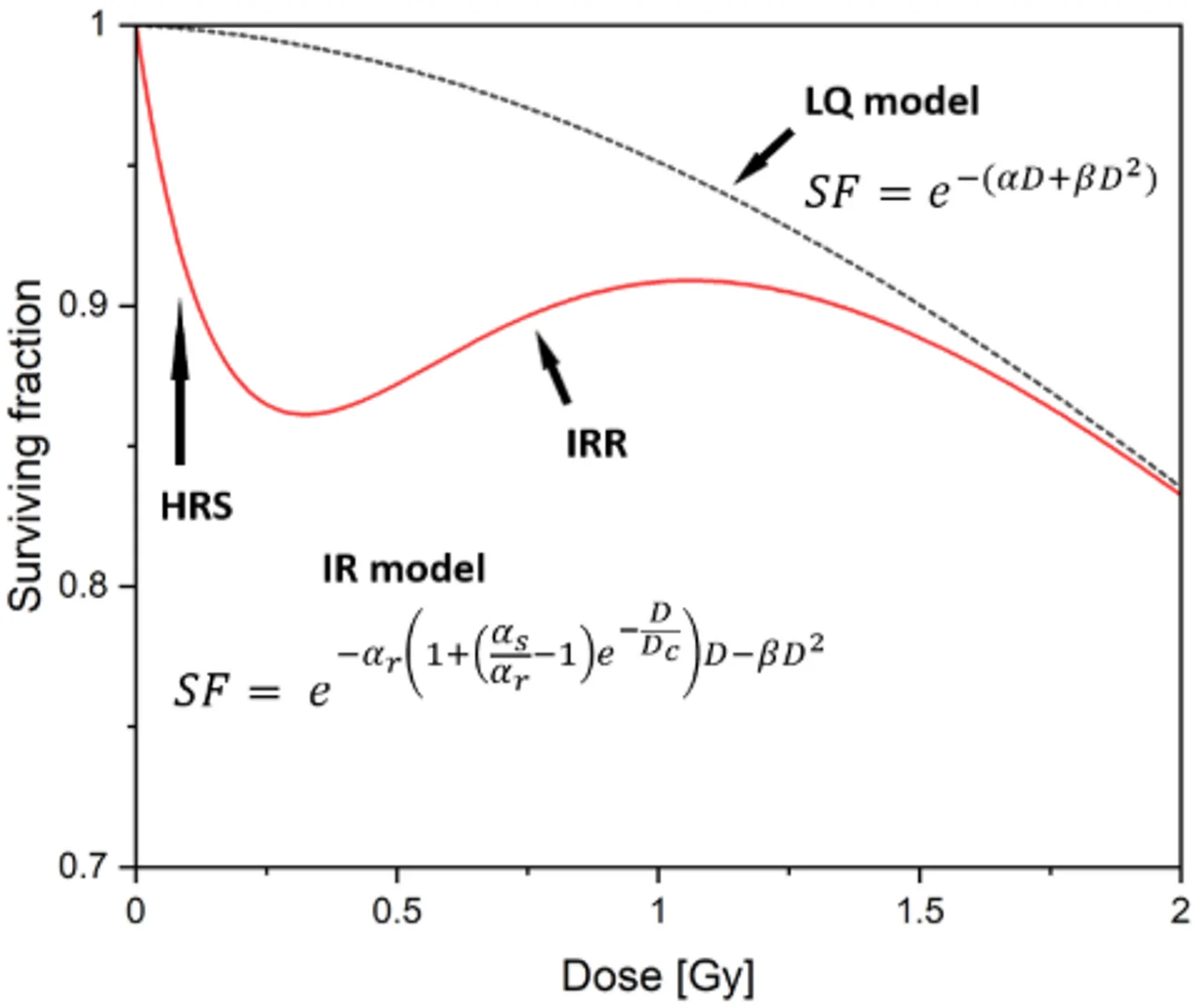
The phenomena of low dose hyper-radiosensitivity and induced radioresistance, as reflected in cell survival curves. These result in a deviation from the linear quadratic (LQ) model and are often described by the induced repair (IR) model. The equations for both models are also shown.

For some cell lines, however, the surviving fraction significantly deviates from the LQ model at low doses (< 1 Gy)^10,11^. These cell lines show a more pronounced decrease in cell survival at very low doses (~ 0.1 Gy) followed by an apparent increase in survival at slightly higher doses (0.3–0.7 Gy). These phenomena are known as hyper-radiosensitivity (HRS) and induced radioresistance (IRR), respectively (Fig. 1). The Induced Repair (IR) model was introduced by Joiner and Johns^12^ as a four-parameter mathematical model to describe this distinct behaviour. According to the model, the surviving fraction can be calculated by the following equation:

2 $$\:SF=\:{e}^{-\left({\alpha\:}_{r}+\left({\alpha\:}_{s}-{\alpha\:}_{r}\right)*{e}^{\left(-\frac{D}{{D}_{c}}\right)}\right)*D-\beta\:*{D}^{2}}$$

where *α*_*r*_ and *β* parameters correspond to the α and β parameters from the LQ model, respectively, *α*_*s*_ represents the initial slope of the curve at very low doses, *D* is the applied dose, and *D*_*c*_ is the dose at which the transition from α_s_ to *α*_*r*_ is 63% complete.

The purpose of this study is to explain the effects of low-dose HRS and IRR by applying the principle of mutation minimisation and cancer risk prevention to the system. To this end, a hypothesis has already been developed^13^, according to which HRS and IRR are the results of an optimisation process aimed at reducing the total number of mutations in the tissue. Cells with the most DNA damage are removed to prevent genomic instability, and a neighbouring cell divides to maintain the number of cells in the tissue. That earlier model demonstrated that selective removal of damaged cells could minimise mutation burden, but it assumed idealised knowledge of mutation levels across the tissue and did not provide a biological mechanism. In the present work, we propose a refined approach—the Minimum Mutation Load (MML) model—in which intercellular communication enables tissues to approximate this optimum through biologically plausible decision-making at the level of individual cells, based on their local environment.

To validate our MML model, we fitted it to clonogenic survival data from numerous in vitro experiments exhibiting HRS and IRR. These datasets were previously compiled into a publicly available database^14,15^. The Induced Repair (IR) model, which is frequently applied to fit such data in published studies, was used as a reference for comparison to evaluate the performance of the MML model.

## Methods

A mathematical model was developed in which two sources of mutations are distinguished: spontaneous mutations occurring during cell division and mutations arising from DNA damages induced by environmental stressors such as ionising radiation. To minimise the number of mutations in a tissue, the elimination of cells with a relatively high number of mutagenic damages can be beneficial. There is a trade-off, however, because the replacement of dead cells results in an increase in cell divisions, and so in spontaneous mutations. As a result, a cell decides whether it survives or dies based on its number of mutagenic damages and the average number of mutagenic damages in its neighbourhood.

It is important to note that mutations and mutagenic damages are conceptually distinct. Mutations refer to permanent changes in the DNA sequence, including point mutations, insertions, deletions, or other alterations that cannot be recognized by the cell and are passed on during replication. They encompass any deviation from the original genetic code. In contrast, mutagenic damage refers to initial structural alterations in the DNA—such as complex double-strand breaks, oxidative base modifications or clustered lesions—that can be recognized and processed by the cell’s DNA damage response mechanisms. These damages are not mutations themselves, but they have the potential to result in mutations if misrepaired or left unrepaired.

In the context of the MML model, mutagenic damage is used as a proxy for the potential mutational burden, and not a direct cause of cell death. It is distinct from lethal damage, which would immediately compromise cell viability. The model does not attempt to mechanistically distinguish between repair outcomes; instead, it treats mutagenic damage as a quantifiable input that influences a cell’s decision to undergo survival or death, based on its relative mutation burden compared to its neighbours. This abstraction enables us to explore the broader hypothesis that tissues may engage in mutation load minimization as a cooperative strategy, without modelling the specific molecular pathways of DNA damage recognition, DNA damage repair, and apoptosis. As the MML model does not consider intracellular processes like the model of Matsuya et al.^16^, it cannot be regarded as mechanistic in the same sense, although as discussed later, it provides a potential mechanism for intercellular communication leading to survival curves showing HRS and IRR, representing a substantial advance over our previous model^13^.

The number of mutagenic damages induced by ionising radiation follows a Poisson distribution over the cells. The average of the Poisson distribution is directly proportional to absorbed dose, with a characteristic induction rate denoted by *µ*. This parameter describes how many mutagenic damages are induced on average by 1 Gy absorbed dose, thus its unit of measure is Gy^− 1^. The value of this parameter in theory could be any positive number, but based on the analysis presented later, it usually falls between 0 and 2 Gy^− 1^. Although the distribution of DNA damages depends on radiation quality^17^, we did not distinguish between low-LET and high-LET radiation in the present work in order to avoid overparameterisation; this aspect was addressed separately in a subsequent extension of the MML model incorporating LET dependence through the Local Effect Model^18^.

The spontaneous mutation rate—i.e. the average number of mutations occurring during cell division—is denoted by *m*_*s*_. These mutations are related to copying errors during cell division. The spontaneous mutation rate is considered to be constant independently of absorbed dose. This parameter is unitless and its value usually falls between 0 and 1 based on the analysis presented later.

The minimization of mutations in a tissue or cell culture system requires communication between the cells. In our computational model, cells are placed on a circle representing a culture plate. It was assumed that each irradiated cell emits a chemical signal, whose strength is proportional to the number of mutagenic damages of the irradiated cell and decreases with the distance according to a Gaussian distribution, reflecting that molecules in water move according to the Brownian motion^19^. Each cell perceives the resultant signal and compares it to its own mutagenic damage number. The inclusion of distance-dependent signalling via physicochemical processes represents a significant improvement compared to our previous model^13^, where the distance between cells was not considered.

Mathematically, the signal strength can be described by the following function:

3 $$\:p=\frac{1}{\sqrt{{\pi\:2\sigma\:}^{2}}}{e}^{-\frac{{x}^{2}}{{2\sigma\:}^{2}}}$$

where *σ*^*2*^ is the variance and *σ* is the half width of the Gaussian distribution, while *x* is the distance from the centre of the signal-emitting cell. The parameter *σ*^2^ determines the effective communication range. The signal strength sensed by cell *i* is denoted by *c*_*i*_, and is calculated by summing the contributions from all the other cells (*j* = 1, …, *N*, except *j* = *i*), i.e.

4 $$\:c_{i} = \sum \:_{{\begin{array}{*{20}c} {j = 1} \\ {\:j \ne \:i} \\ \end{array} }}^{N} G_{{ij}} \cdot \:L_{j}$$

where *L*_*j*_ is the number of mutagenic damages in cell *j*, *G*_*ij*_ are elements of a symmetric matrix derived from Eq. (3), using the distance between cells *i* and *j* as *x*, and *N* is the total number of cells in the system. A cell dies if the number of its mutagenic damages exceeds the sum of the spontaneous mutation rate and the average number of mutagenic damages in its neighbourhood. This occurs when it is expected that removing the cell will reduce the total number of mutations in the system, even accounting for the additional division required to replace it. Mathematically, this is defined by the inequality:

5 $$\:{m}_{i}>\frac{{c}_{i}}{{c}_{0}}+{m}_{s}$$

where *m*_*i*_ is the number of mutagenic damages in cell *i*, *c*_*0*_ is a normalization factor ensuring that *c*_*i*_ / *c*_*0*_ reflects the local average number of mutagenic damages. *c*_*0*_ was determined by calculating the average *c*_*i*_ if all the cells had one mutagenic damage. Since this value depends on the spatial arrangement of cells (i.e. the cell configuration), the average was computed over all randomly generated configurations used in the simulations.

In the MML model, cell death is triggered by relative damage. Cells are eliminated only when their own mutagenic damage exceeds the average damage level inferred from their local neighbourhood plus the mutation cost of replacement. Therefore, if all cells have identical damage, no cell is an outlier relative to its neighbours, and therefore no selective elimination occurs.

In its present form, the model does not explicitly include time as an independent variable. This simplification was made deliberately in order to avoid overparameterisation while focusing on the relationship between local damage burden and survival outcome. Accordingly, damage evaluation and the associated cell fate decision are treated as occurring within a single post-irradiation decision step. This approximation introduces a limitation: while the damage signal is described by a Gaussian spatial distribution, the model does not address the dynamic processes by which such a distribution emerges through signal emission, diffusion, degradation, and cellular response kinetics.

In this study, the signalling mechanism is considered paracrine, meaning that each cell responds only to signals from neighbouring cells, not to its own. The inclusion or exclusion of autocrine signalling, however, has no effect on the surviving curves. As the normalization factor *c*_*0*_ increases proportionally to the signals received by the cells, adding an autocrine term rescales both numerator and denominator equally, leaving the normalised response unchanged.

Simulations were performed as follows. First, *N* = 600 cells were placed randomly from a uniform distribution over a circle with a radius of *R* = 0.5, ensuring a minimum distance of *d* = 0.006 between the cells’ centres, which reflects the diameter of a cell. Pairwise distances were then computed, and the matrix *G* was calculated for *σ*^*2*^ = 0.01. This value ensures that intercellular communication is neither too localised nor too global: the signal strength at a cell’s edge is approximately 60%, and each cell receives around 95% of its total signal from within a 0.25 *R* neighbourhood. To assess uncertainties, 100 independent simulations were conducted for each dose, using 100 different cell configurations. While the model is independent of the physical size of the system, these unitless parameters correspond to the following parameter set in SI units: if the cell diameter *d* is 10 μm, then σ ≈ 170 μm, *R* ≈ 1 mm, resulting in a cell density of around 190 cell mm^− 2^.

Since this is the first presentation of this model, we aimed to exclude boundary effects. Cells positioned near the edge of a circular domain may receive fewer signals due to the reduced number of neighbouring cells. To address this, cells were randomly placed on a larger circle with radius 2R, maintaining the same cell density as in the original configuration. Surviving fractions—defined as the ratio of surviving cells to total cells—were calculated only for those located within the central circle of radius R, while signals from all cells on the larger circle (radius = 2R) were included in the computation.

As the model cannot be described by an analytical function, numerical methods were needed to fit model parameters to experimental data. For this purpose, the Nelder–Mead algorithm^20^, a simplex minimisation method, was used to optimise the values of the spontaneous mutation rate *m*_*s*_, and mutagenic damage induction rate *µ*. The Nelder–Mead fitting method is a direct search technique for nonlinear optimisation problems in which derivatives are unknown and is thus suitable for models without a defined analytical form.

For a given experimental dataset, the Nelder–Mead algorithm determines the optimal values of *m*_*s*_ and *µ* by minimising the difference between the experimental surviving fractions and those predicted by the simulations at the corresponding dose points. The method uses the concept of the simplex in two dimensions and takes into account the uncertainties of the experimental data. The optimisation begins with three parameter pairs forming a triangle in the parameter space. In each iteration, the algorithm evaluates possible improvements by applying a series of tests—reflection, expansion, and contraction—based on the centroid of the line connecting the two vertices with smaller differences. If a test yields a new vertex with a smaller difference than the current worst, it replaces it; otherwise, the simplex is shrunk, and a new iteration begins. In the present work, the process terminates when either the difference is reduced below the selected threshold of 0.1, or the selected maximum of 50 iterations is reached.

It is important to note that surviving fractions are not expected to be governed by mutation minimisation at high doses, where other biological processes dominate cell death^21^. Indeed, cells may be unable to survive if they have too many DNA damages. Therefore, it was assumed that the LQ model accurately describes surviving fractions at high doses, which are not affected by the mechanisms responsible for HRS and IRR at low doses. Reflecting this assumption, the *α* and *β* parameters of the LQ model were determined by fitting the function to experimental data above 1 Gy, or to at least three data points if fewer than three were above 1 Gy. For each dose, the final surviving fraction was defined as the minimum of the values predicted by the Minimum Mutation Load (MML) model and the LQ model.

Experimental data for model validation were taken from our previous study^14^. A total of 99 datasets were compiled from 46 articles published between 1993 and 2021. Fits obtained with our MML model were compared with those obtained using the Induced Repair (IR) model. Adjusted R^2^ values were calculated for both models, and the results were evaluated to determine which provided a better fit to the experimental data.

The origin and classification of the cell lines used were also considered in the analysis. The experiments involved a variety of cell types, with a total of 45 distinct types represented in the database. The most frequently used were T98G, a human brain glioblastoma cell line (13 datasets), V79, a Chinese hamster lung fibroblast line (also 13 datasets), and A549 human lung adenocarcinoma line (8 datasets). Overall, 79 experiments featured human-derived cell lines, 16 used Chinese hamster cell lines, and 4 involved rat-derived cell lines.

In line with standard practice for clonogenic survival assays, the vast majority of these cell lines were immortalized, allowing for extended proliferation and consistent colony formation. Although further biological stratification (e.g., based on tumorigenicity or contact inhibition) would be valuable, such information is not consistently reported in the original studies^22^ and could not be reliably assigned across all datasets. Additional characteristics—including cell cycle arrest or unique experimental treatments—are documented where available in the accompanying database.

In the clonogenic assay, surviving fractions are normalized to the plating efficiency of the unirradiated control^4^, meaning that the surviving fraction at 0 Gy equals 1 by definition. Among the datasets we analysed, there were only three from the same publication^23^ that reported values at 0 Gy different from 1, while in several datasets the surviving fraction at 0 Gy was not reported. To ensure consistency, all survival curves were treated according to the standard clonogenic assay, and the surviving fraction at 0 Gy was assumed to be 1. In the exceptional cases where the surviving fractions at 0 Gy differed from 1, the data were renormalized to *SF* at 0 Gy. Although the model could be modified to include an additional scaling factor allowing *SF*(0) < 1, this was not implemented in the present analysis, as such a parameter would primarily affect normalization rather than the low-dose hyper-radiosensitivity and induced radioresistance behaviour of interest.

## Results

### Relationships between model parameters and characteristics of the survival curve

**Fig. 2:**
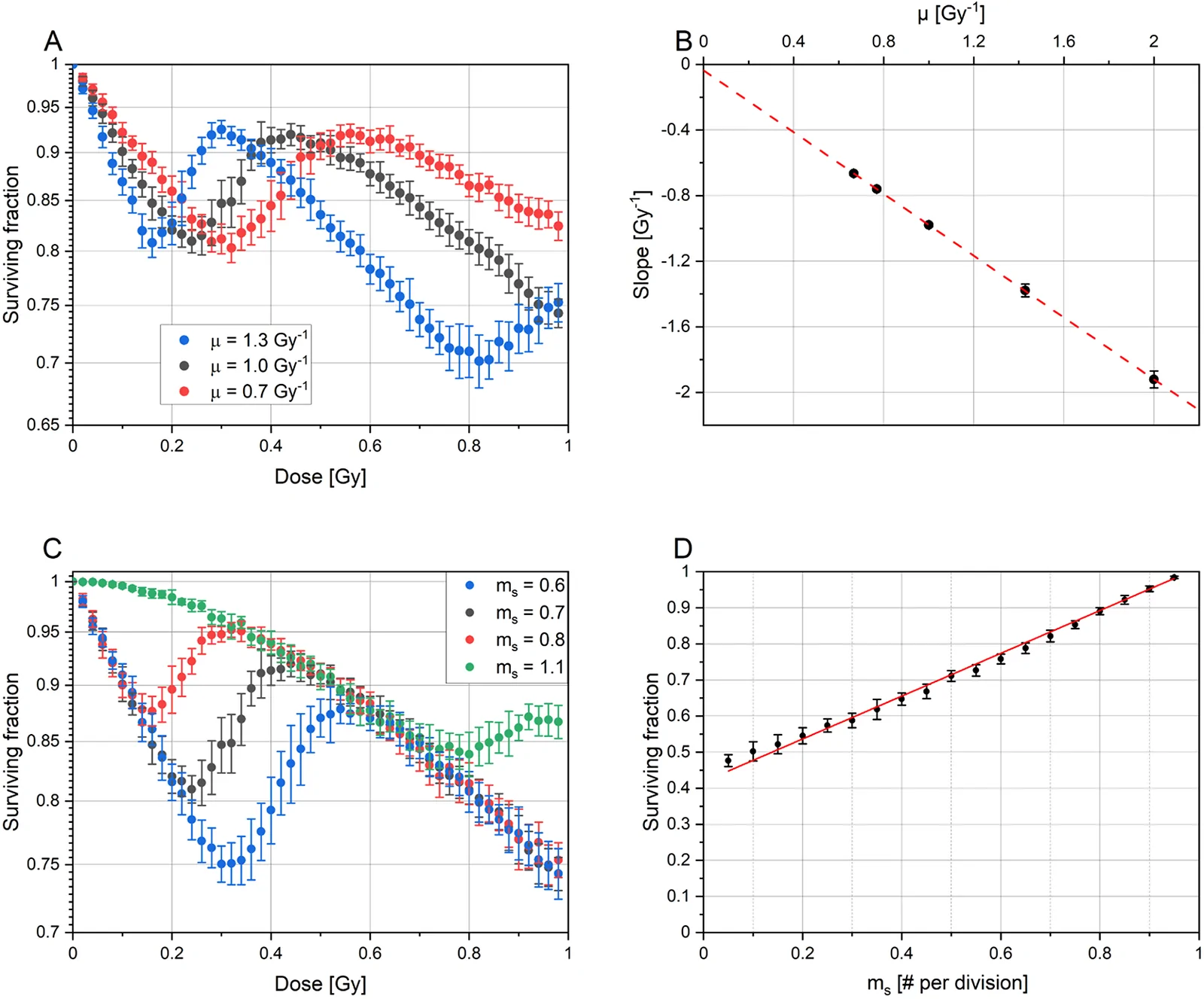
The dependence of survival curves on the mutagenic damage induction rate (µ) and the spontaneous mutation rate (m_s_), when the other parameter is fixed. In panel **A**, m_s_ = 0.7, while µ is 1.3 (blue), 1.0 (black) or 0.7 Gy^-1^ (red). As µ increases, the local minimum shifts to higher doses. A second local minimum can be seen in case of µ = 1.3 Gy^-1^ at 0.8 Gy. In panel **B**, the initial slope of the survival curve is plotted as a function of µ if m_s_ = 0.7. In panel **C**, µ = 1 Gy^-1^, while m_s_ is 0.6 (blue), 0.7 (black), 0.8 (red), or 1.1 (green). As m_s_ increases, but remains below 1, the surviving fraction at the local minimum becomes higher and shifts to lower absorbed doses. Panel **D** shows the surviving fraction at the local minimum as a function of m_s_ if it is lower than 1. These linear relationships are used to initialise parameter values when fitting to experimental data.

First, we focus on how the surviving fraction depends on the mutagenic damage induction rate *µ*, and the spontaneous mutation rate *m*_*s*_. Figure 2 shows survival curves for three different values of *µ* with *m*_*s*_ fixed at 0.7 (Fig. 2A), and survival curves for four different values of *m*_*s*_ with *µ* fixed at 1 Gy^− 1^ (Fig. 2C). The figure shows clear patterns for both parameters.

The mutagenic damage induction rate *µ* determines the initial slope of the survival curve (Fig. 2A). The higher *µ* is, the steeper the initial slope. If *m*_*s*_ is between 0 and 1, then a more precise statement can be made: the initial slope is linearly proportional to *µ* (Fig. 2B). As *µ* increases, the absorbed doses at which the local minimum and maximum occur shift to higher values, and the gap between them also increases. The surviving fractions at the local minimum and maximum, however, remain unaffected.

The spontaneous mutation rate *m*_*s*_ determines the surviving fractions at the local minimum and maximum (Fig. 2C). The higher *m*_*s*_ is, the higher the surviving fractions at both points (Fig. 2C). The difference between the surviving fractions at the local minimum and maximum decreases with increasing *m*_*s*_ (Fig. 2C). Indirectly, *m*_*s*_ also influences the absorbed dose at which the local minimum and maximum occur: as *m*_*s*_ increases, both shift to lower doses. However, *m*_*s*_ does not affect the initial slope of the survival curve if it is lower than 1. While *m*_*s*_ can also be higher than 1 (see Fig. 2C), it was already discussed in ^13^, and did not provide a better fit in the present analysis of experimental data.

**Fig. 3:**
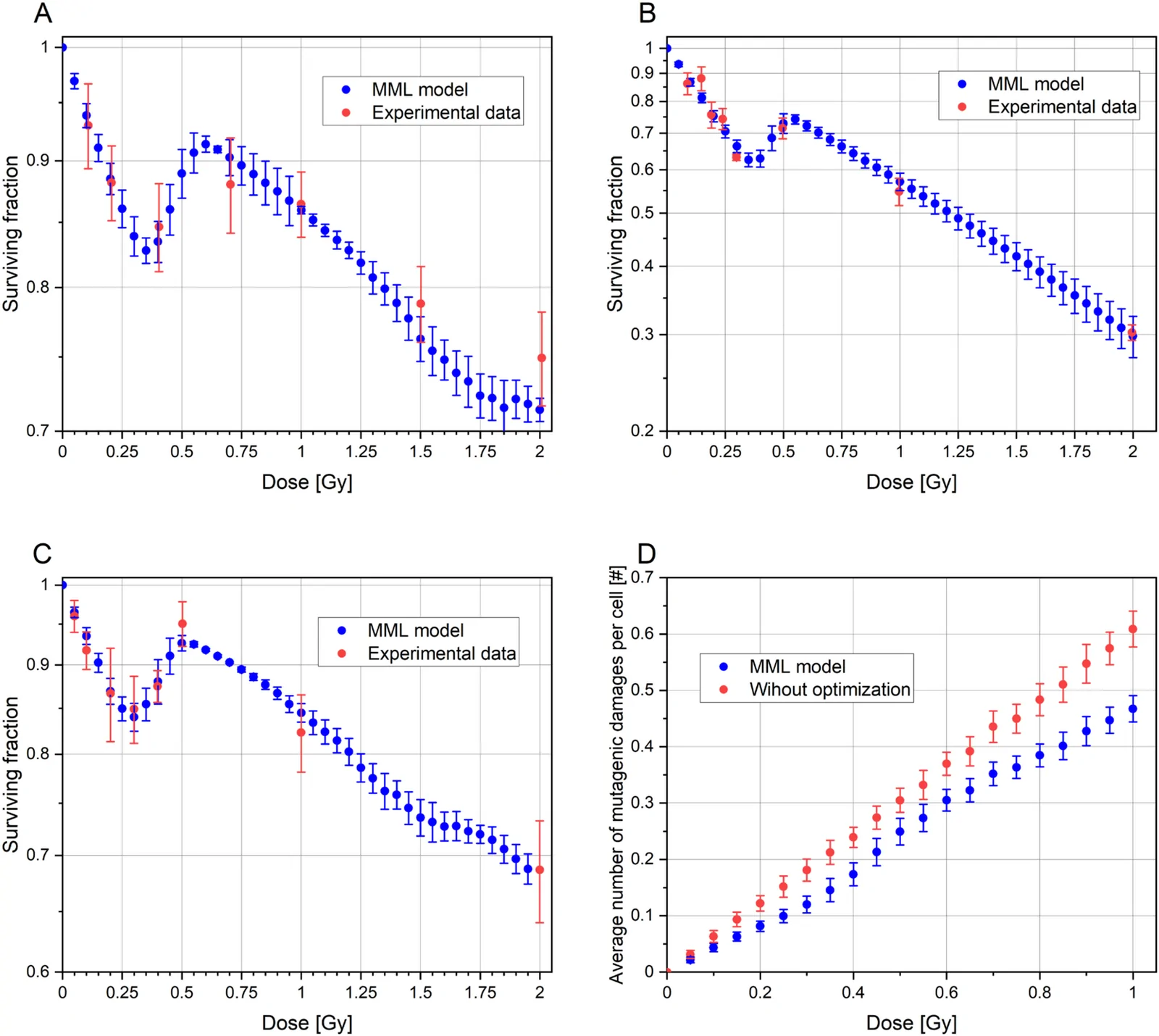
Examples of successful fits to experimental data. Experimental datasets were taken from Short et al.^24^(panel **A**, dataset ID: 17 in^15^), Słonina et al.^25^(panel** B**, dataset ID: 81 in^15^), and Dai et al.^26^ (panel **C**, dataset ID: 60 in^15^). Panel **D** shows the mutation load as a function of dose using the parameters obtained for experimental data in panel **C**. The mutation burden is substantially higher when selective removal is disabled (red dots), compared to the MML model with selective removal (blue dots). The slight deviations from the LQ model around 1.8 Gy in panel **A** and around 1.6 Gy in panel C are related to the second local minimum of the MML model.

It is also worth noting that there are multiple local minima in the surviving curves (see the blue curve in Fig. 2A). Similarly to the case of *m*_*s*_ greater than 1, it was discussed in ^13^. However, their existence does not play a major role in the present analysis of experimental data. In most cases, the LQ model results in lower surviving fractions at the doses, where these second local minima can be observed. In some cases, however, a second local minimum results in a slight deviation from the LQ model, e.g. in Fig. 3A and C.

### Background of the relationships between model parameters and survival curve characteristics

To understand why survival curves depend on the model parameters in the way described above, the terms in Eq. 5 must be considered. At very low doses, the average number of mutagenic damages in the neighbourhood of most cells—and therefore the relative signal strength (c_i_ / c₀)—is very low. As a result, cells with any mutagenic damage will die if *m*_*s*_ is lower than 1. If *m*_*s*_ is higher than 1, the survival curves differ markedly^13^. As the number of cells with mutagenic damages is directly proportional to the average number of mutagenic damages at very low doses, which is proportional to absorbed dose, the initial slope of the survival curve is also directly proportional to the absorbed dose.

As the average number of mutagenic damages increases, the signal received by the cells also increases. For any given value of the spontaneous mutation rate, there is a value of relative signal strength (*c*_*i*_/*c*_*0*_)—and a corresponding absorbed dose—at which the right-hand side of Eq. 5 equals one. At this point, cells with one mutagenic damage in such a neighbourhood no longer die. This absorbed dose marks the point, at which the survival curve begins to increase. A higher spontaneous mutation rate lowers the required signal strength and average number of mutagenic damages needed for a given cell to survive, leading to a higher surviving fraction at the local minimum.

Understanding the relationship between model parameters and the properties of the survival curves is essential for fitting the model to experimental data, since the model cannot be expressed as an analytical function. As shown in Fig. 2, the model can generate a wide variety of survival curves by adjusting these two parameters. Moreover, the linear relationships between the model parameters and survival curve characteristics (Fig. 2B and D) allow us to estimate initial parameter values from experimental data, which are then used to initialise the Nelder-Mead optimisation procedure.

### Determining initial values of model parameters from experimental data

The initial value of *µ* is determined from the initial slope of the experimental survival curve. In practice, a linear function was fitted to the first two, three, four, and five data points, taking their uncertainties into account. If the uncertainty at 0 Gy was reported as zero (as in most cases), the fitted function was constrained such that *SF* (0 Gy) = 1. The negative of the slope estimate with the lowest associated uncertainty was selected as the initial value for *µ*.

The initial value of *m*_*s*_ was determined using the surviving fraction at the local minimum of the experimental data. The local minimum of the survival curve was identified for multiple values of *m*_*s*_, and the corresponding values were plotted in Fig. 2D. This analysis showed a linear relationship between the local minimum and *m*_*s*_. Fitting a linear function to these simulated data (for the specific model setup: *N* = 600, *R* = 0.5, *σ*² = 0.01, *c₀* = 196) yielded the following equation, which can be used to estimate the initial value of the spontaneous mutation rate:

6 $$\:{m}_{s}=\frac{{SF}_{lm}-0.4257(\pm\:0.0067)}{0.5671(\pm\:0.0117)}$$

where *SF*_*lm*_ is the surviving fraction at the local minimum of the experimental data. The standard deviations of the fitted coefficients are shown in parentheses.

### Fitting the model to experimental data

A total of 100 independent simulations were performed for each parameter set. These parameters were obtained using the initial estimates and the Nelder–Mead algorithm, as described in the Methods section. Since the surviving fraction depends on the spatial configuration of cells, 100 different configurations were used. This variability contributes to the uncertainty of the surviving fractions, and consequently to the uncertainty of the fitted model parameters. The simulated results were compared to the experimental data using the adjusted R² metric. In most cases, good agreement with experimental data was achieved by adjusting *µ*, *m*_*s*_ and the LQ model parameters *α* and *β*. Representative examples are shown in Fig. 3.

While the model produced good fits in most cases, this was not always true. In some cases, the Nelder–Mead method converged on a local, rather than global, minimum, resulting in suboptimal parameter estimates. Two examples are presented below in which small adjustments to the fitting procedure improved the outcome.

**Fig. 4:**
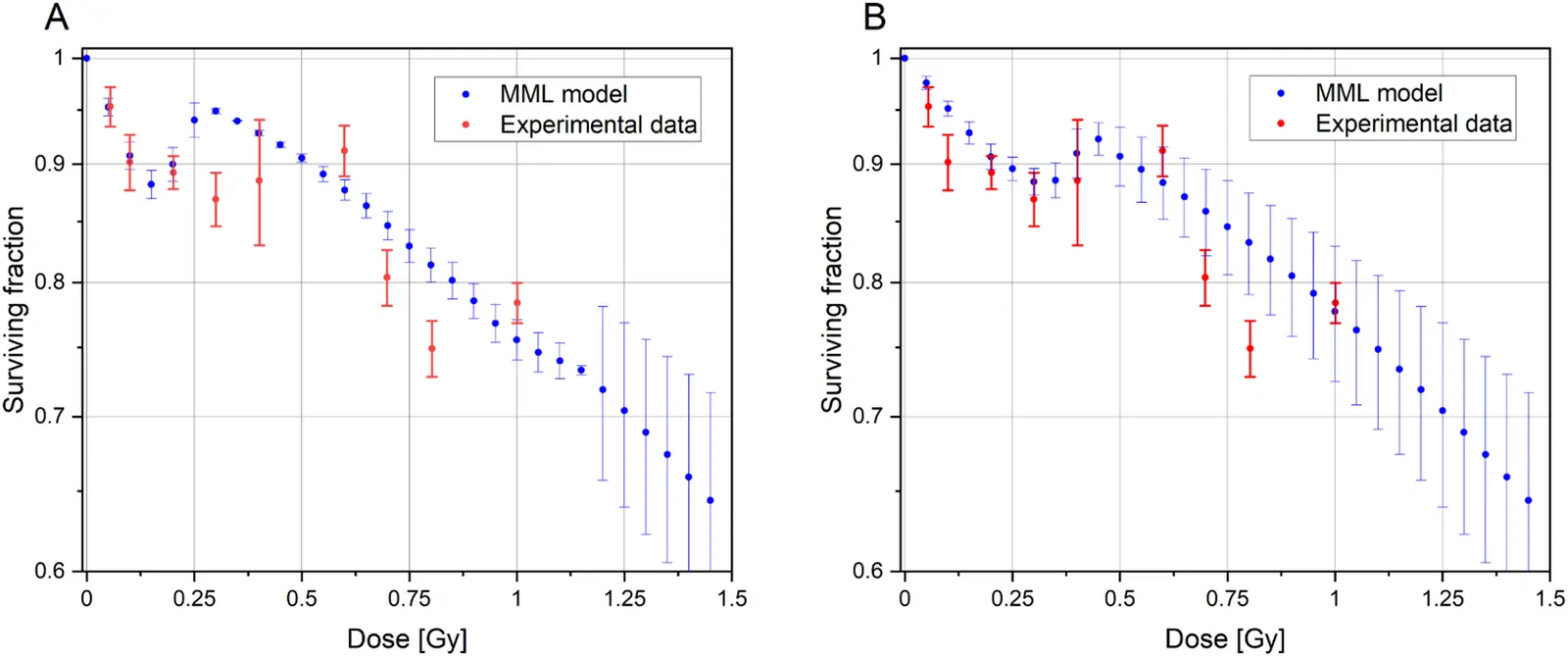
Example of non-optimal automatic fitting to experimental data of Wykes et al.^27^ (dataset ID: 49 in ^15^). The first two experimental data points suggest an initially steep drop in survival, leading the model to result in a local maximum at a dose too low. The sharp increase in uncertainty of the simulated data at high doses arises from large uncertainties in the LQ parameters. Panel **B** shows the corrected fit where the manually corrected µ results in a better fit. The R^2^ values of the fitting in panels **A** and **B** are 0.91 and 0.98, respectively. For better visibility of the low-dose region, experimental data above 1.5 Gy are not displayed, but they were considered during the fitting procedure.

One common issue arose from the method used to determine the initial value of *µ*. If one of the lowest-dose data points deviates from a linear trend, the simulated curve may initially undershoot (if the deviating experimental surviving fraction is too low) or overshoot (if the deviating experimental surviving fraction is too high) relative to the experimental curve. Examples of this effect are shown in Fig. 4. In such cases, better results can be obtained by selecting a different initial *µ* value—even if it does not correspond to the slope estimate with the lowest uncertainty.

**Fig. 5:**
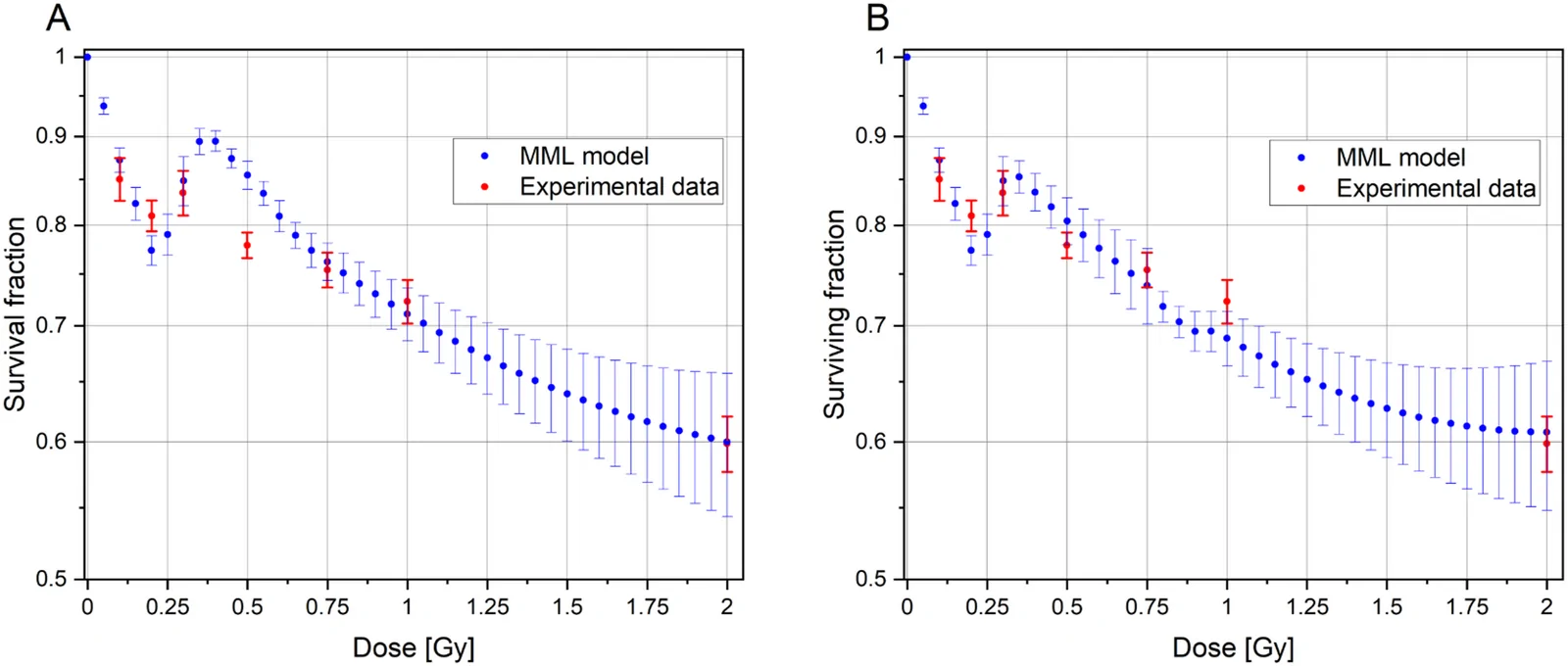
Example of non-optimal automatic fitting to experimental data of Edin et al.^28^ (dataset ID: 59 in ^15^). In panel **A**, only the last three data points were used to fit the LQ model, leading to an overestimation of the local maximum. Panel **B** shows the corrected fit by obtaining the LQ model parameters by taking into account surviving fraction values below 1 Gy. The R^2^ values of the fitting in panels A and B are 0.89 and 0.95, respectively.

Another source of error is the poor fitting of the LQ model due to the insufficient number of data points above the 1 Gy threshold. In such cases, extrapolation leads to inaccurate survival curve estimates at lower doses. This can be addressed by including additional points below 1 Gy when estimating *α* and *β*. Figure 5 illustrates this correction.

**Fig. 6:**
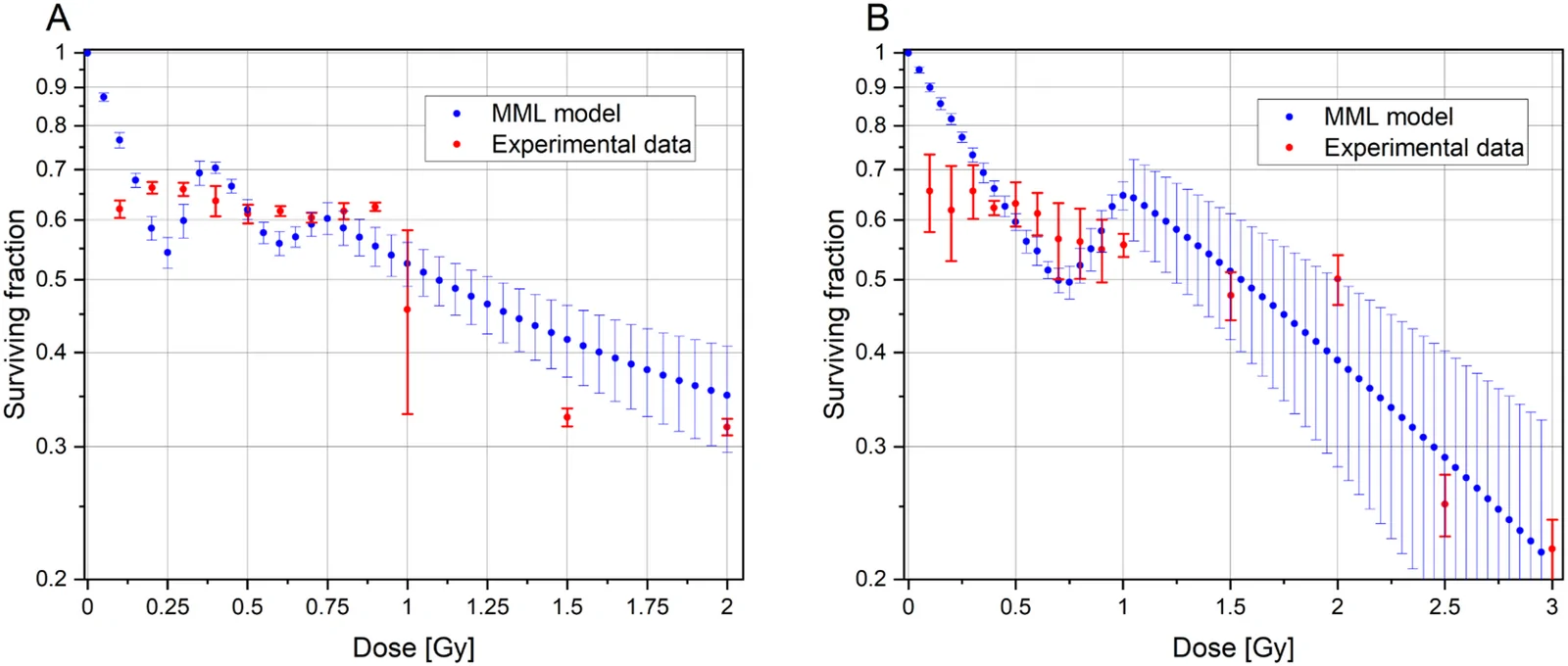
Two different examples of poor fits to the data from Krueger et al.^29^. In panel **A**, the difference between the surviving fractions at local minimum and maximum is much larger in the simulated curve than in case of the experimental data (dataset ID: 65 in^15^). In panel **B**, the experimental survival curve (dataset ID: 63 in^15^) has a plateau instead of showing significantly different local minimum and maximum, which cannot be fitted with the model parameters.

Beyond these procedural corrections, there are datasets for which the model fails to reproduce the survival curve by adjusting only *µ* and *m*_*s*_. For example, when the difference between the survival fraction at the local minimum and maximum is low—and the minimum itself is low—the model tends to overestimate this difference (Fig. 6A). This is because a low local minimum corresponds to a small value of m_*s*_, which results in a larger difference between the surviving fractions at the local minimum and maximum. In other cases, the survival curve is almost flat, with no clear minimum or maximum (Fig. 6B), making it incompatible with the assumptions of the MML model. Although changes in cell density might improve the fit in such cases, these data are rarely provided in the publications, and we chose to avoid overparameterisation of the model.

### Overview of the goodness of fits considering a large database

**Fig. 7:**
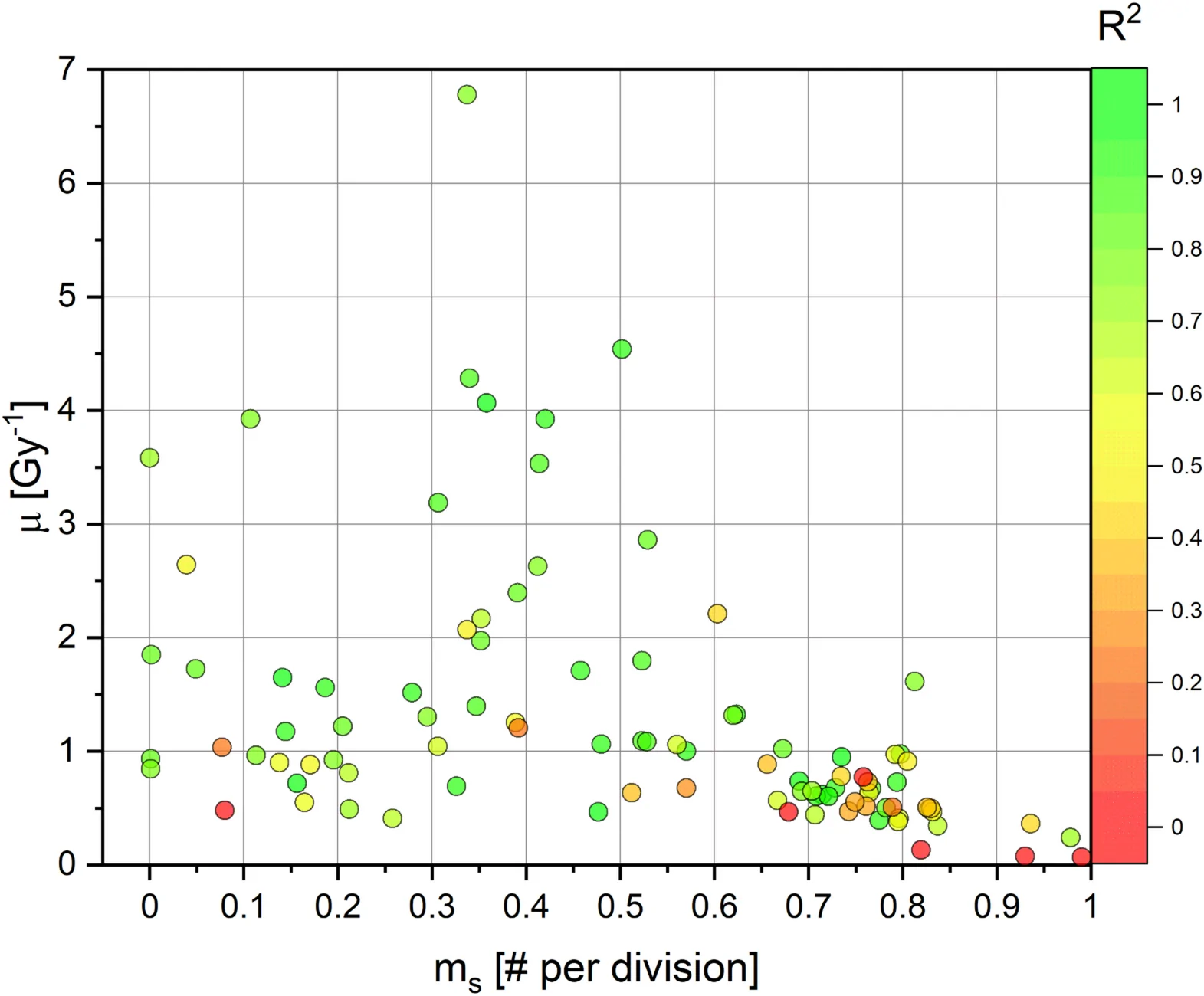
The fitted m_s_ – µ parameter pairs for all experimental data. The colour of the circle indicates the goodness of the fit according to the adjusted R^2^ method.

Overall, the model was fitted to 99 experimental datasets obtained from the previously published experimental database^14^. The generated *m*_*s*_ - *µ* parameter pairs of the completed fits are compiled and shown in Fig. 7. The goodness of fit was assessed using the adjusted R² metric, which is represented in Fig. 7 by the colour of the circles.

The average adjusted R^2^ was 0.74, with the standard deviation of 0.19. In 42 cases, the fit exceeded 0.8, which was considered as a relatively good fit. In 11 cases, poor fits were attributable to dataset characteristics similar to those discussed earlier and illustrated in Fig. 6. In these cases, the adjusted R² was negative—that is, the fit performed worse than a constant function. In case of datasets with negative R^2^, we checked whether using the LQ model alone would provide a better fit, but such improvement was not observed.

**Fig. 8:**
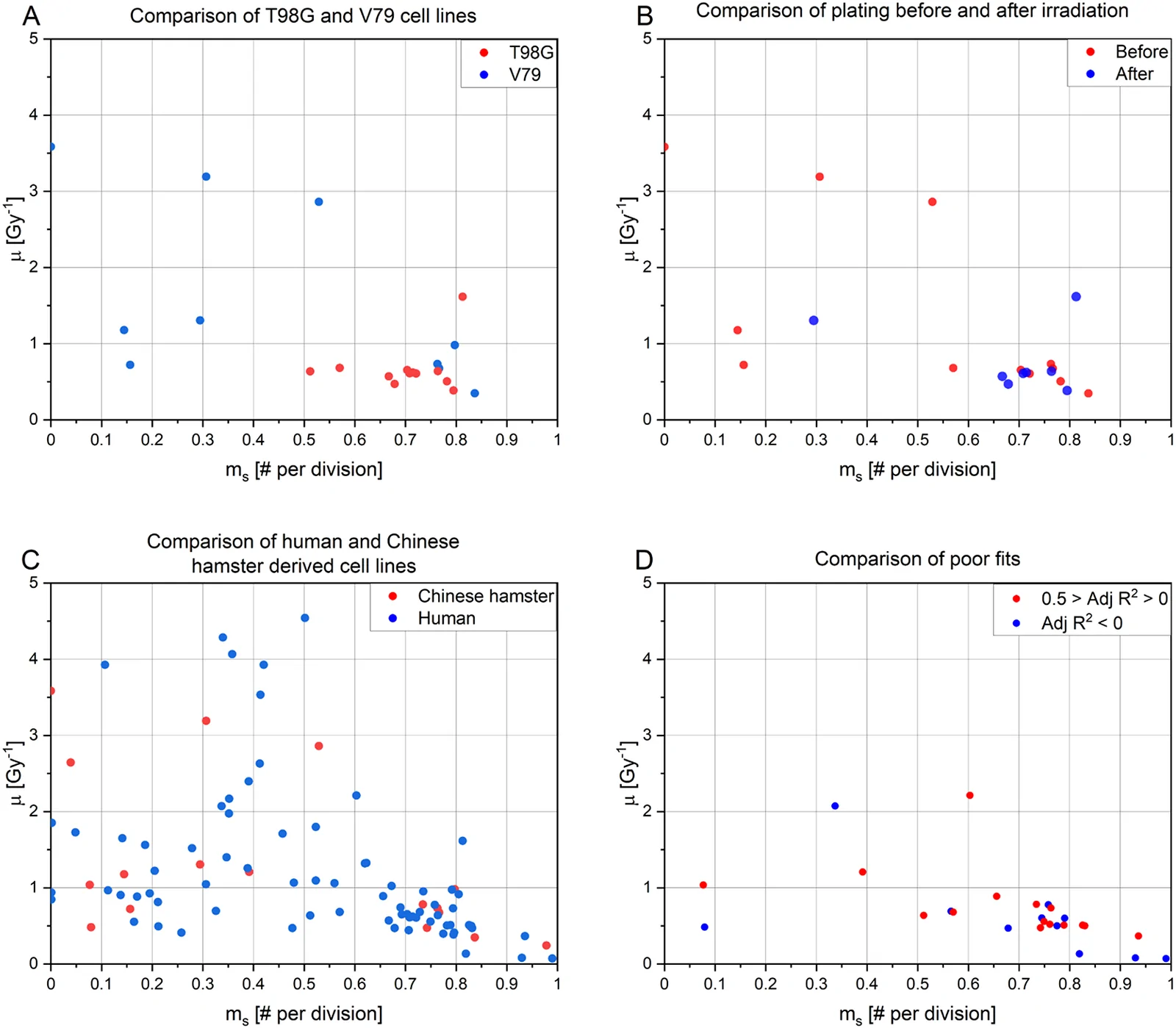
Fitted m_s_ – µ parameter pairs for experimental datasets for different characteristics: panel **A,** for the two most frequently used cell lines, T98G (red) and V79 (blue), each with 13 datasets; panel **B,** for comparison of plating before (red) or after (blue) irradiation for the T98G and V79 cell lines; panel **C,** for comparison of human-derived (blue) and Chinese hamster-derived (red) cell lines; and panel **D,** for datasets with poor model fits with red points corresponding to datasets with adjusted R^2^ between 0 and 0.5, and blue points indicating datasets with negative adjusted R^2^.

The specific characteristics of the individual datasets were also investigated to identify potential biological or methodological trends in the fitted parameters. Among the most frequently represented cell lines, T98G and V79, a clear distinction was observed in the distribution of the fitted parameters µ and *m*_*s*_. Specifically, the T98G cell line exhibited a smaller standard deviation for both parameters (m_s_ = 0.7 ± 0.08; µ = 0.67 ± 0.29) compared to the V79 line (m_s_ = 0.46 ± 0.3; µ = 1.56 ± 1.12) (Fig. 8A). No consistent trend was observed when comparing parameter distributions between human-derived and Chinese hamster-derived cell lines (Fig. 8C). This outcome was expected, given the diversity in cell types, culture conditions, and irradiation protocols across the compiled studies.

Two experimental approaches in colony-forming assay were also compared for T98G and V79 cell lines. Plating before and after irradiation, as defined in Step 9 of the assay protocol by Franken et al.^4^ affects the MML parameters. Notably, datasets using post-irradiation plating showed reduced variability in the fitted µ and m_s_ parameters, suggesting a potential methodological influence on model robustness (Fig. 8B).

To evaluate whether poor model fits were associated with specific biological characteristics, 16 datasets with adjusted R² values between 0 and 0.5 were examined first (Fig. 8D). These datasets represented 12 distinct cell lines from both human and animal origins, covering 9 different tissue types. No consistent trend was observed that would link poor fit quality to species, tissue type, or reported experimental conditions.

Next, 11 datasets with negative adjusted R² values were analysed. These involved 9 distinct cell lines across 6 tissue types. Again, no systematic biological or experimental feature was apparent that could account for the poor model performance. With respect to parameter values, the µ parameter tended to be below average in both categories of poor fits, while no such trend was observed for the m_s_ parameter. The below average of the µ parameter can be attributed to the difficulties to fit to experimental data with steep initial survival and low SF at the local minimum, as it was discussed before.

These findings indicate that, aside from a few limited trends, it is not feasible to draw reliable biological conclusions based on the fitted model parameters. This is due to the considerable heterogeneity in cell line origins, experimental preparation protocols, irradiation conditions, and assay methods. Even among experiments using the same cell line, variations in culture conditions, treatment timing, and cell cycle synchronization contribute to inconsistent outcomes. As standardized experimental setups across studies remain rare, this variability likely obscures any underlying biological trends within the parameter distributions.

**Fig. 9:**
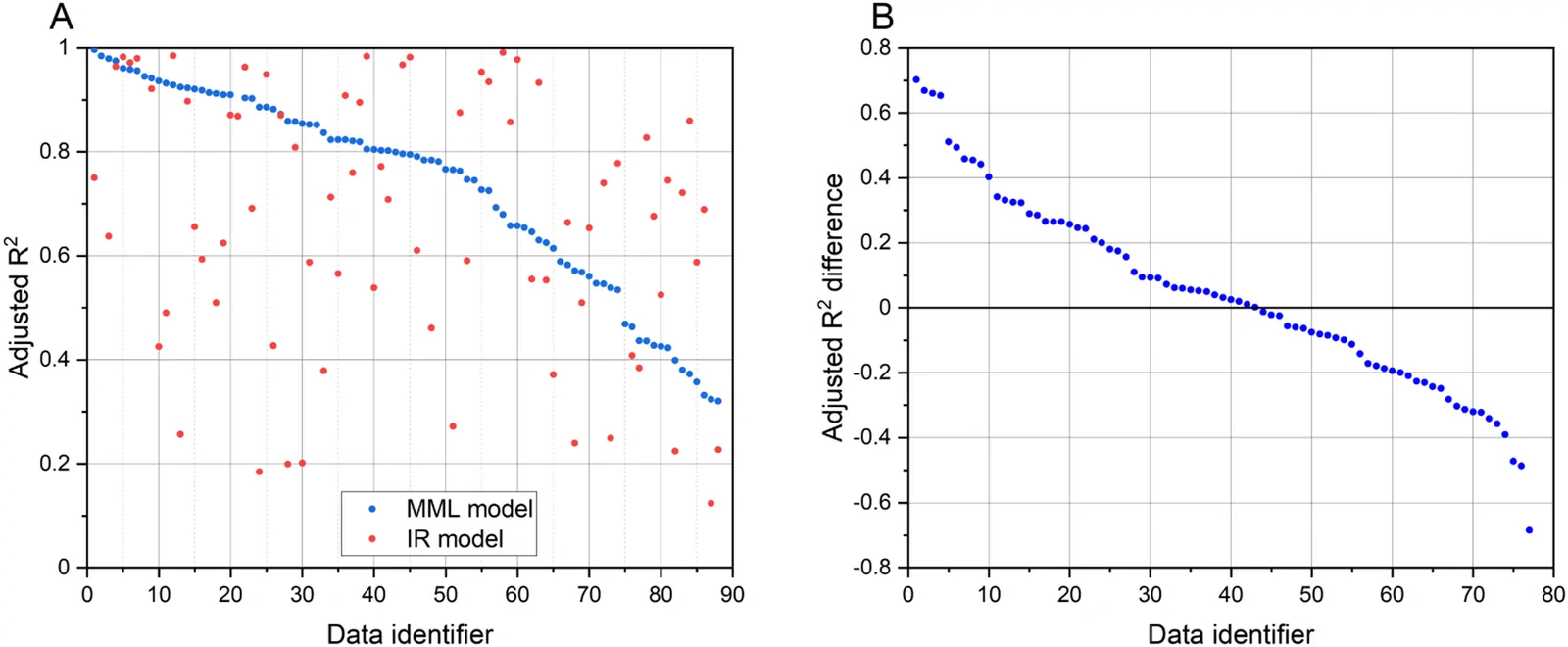
Goodness of fit of the MML model determined by the adjusted R^2^ metric, and comparison with the IR model. Panel **A**: Adjusted R² values for 88 experimental datasets fitted with the MML model (blue) and the IR model (red). Each point on the X-axis corresponds to one dataset, sorted in descending order of the MML model’s adjusted R² for clarity. Panel **B**: Differences in adjusted R² values between the MML and IR models, again sorted in descending order for readability.

For the remaining 88 datasets, the MML model produced a positive adjusted R² value, shown in descending order in Fig. 9A. The mean and standard deviation of the parameters were *µ* = 1.57 ± 1.8 Gy^-1^ and *m*_*s*_ = 0.49 ± 0.27 with the median 0.96 Gy^-1^ and 0.52, respectively.

A comparison with the Induced Repair (IR) model was also performed using adjusted R^2^ values as the metric of fit quality. A direct comparison between the two models was possible for 77 datasets, where both models produced positive adjusted R^2^ values. In this subset, the MML model provided a better fit in 42 cases, while the IR model performed better in 35 cases. The differences in adjusted R² values were generally small, as illustrated in Fig. 9B. In case of the 11 datasets, where the MML model produced a negative adjusted R^2^, the IR model converged in eight cases but did not converge in three cases. In addition, there were 11 datasets where the IR model did not converge, but the MML model produced a positive adjusted R^2^. Overall, these results indicate that the MML model performs comparably to the IR model, which was used here as a benchmark.

## Discussion

Since HRS and IRR may have important implications for cancer therapy, several biophysical models have been proposed to explain the phenomena. One of the earliest hypotheses was that HRS arises from two subpopulations of cells in different cell cycle stages, each with different radiosensitivities^30^. The steep decrease in surviving fraction at low doses reflects death of cells in the more radiosensitive stage, while cells in the more radioresistant phase contribute to stable decrease at moderate and high doses. Although this two-population hypothesis can account for changes in the slope of the survival curves, it cannot explain the increase in survival observed in the IRR region—a key limitation compared to our model. Moreover, this interpretation has been disputed in the broader literature, including in reviews such as that by Joiner et al.^31^, which emphasise alternative explanations involving adaptive responses and changes in DNA repair capacity.

Induced radioresistance is usually linked to activation of additional DNA repair pathways^31,32^ at a given threshold dose that remain inactive at lower doses. This activation can enhance cell survival at moderate doses by improving repair of radiation-induced damage. Nuta et al.^33^, however, concluded that the shoulder region of the survival curve, at doses between 0.5 and 3 Gy, is not caused by increased repair of chromosomal damage. T﻿he MML model explains this region as a result of the mutation minimization mechanism. As written in Eq. 5, cells die only if the neighbouring average damage plus the damage expected during division is smaller than their own mutagenic damage. As the average damage increases with the dose, a threshold emerges where cells with one damage will start to survive, thus increasing the surviving fraction.

**Fig. 10:**
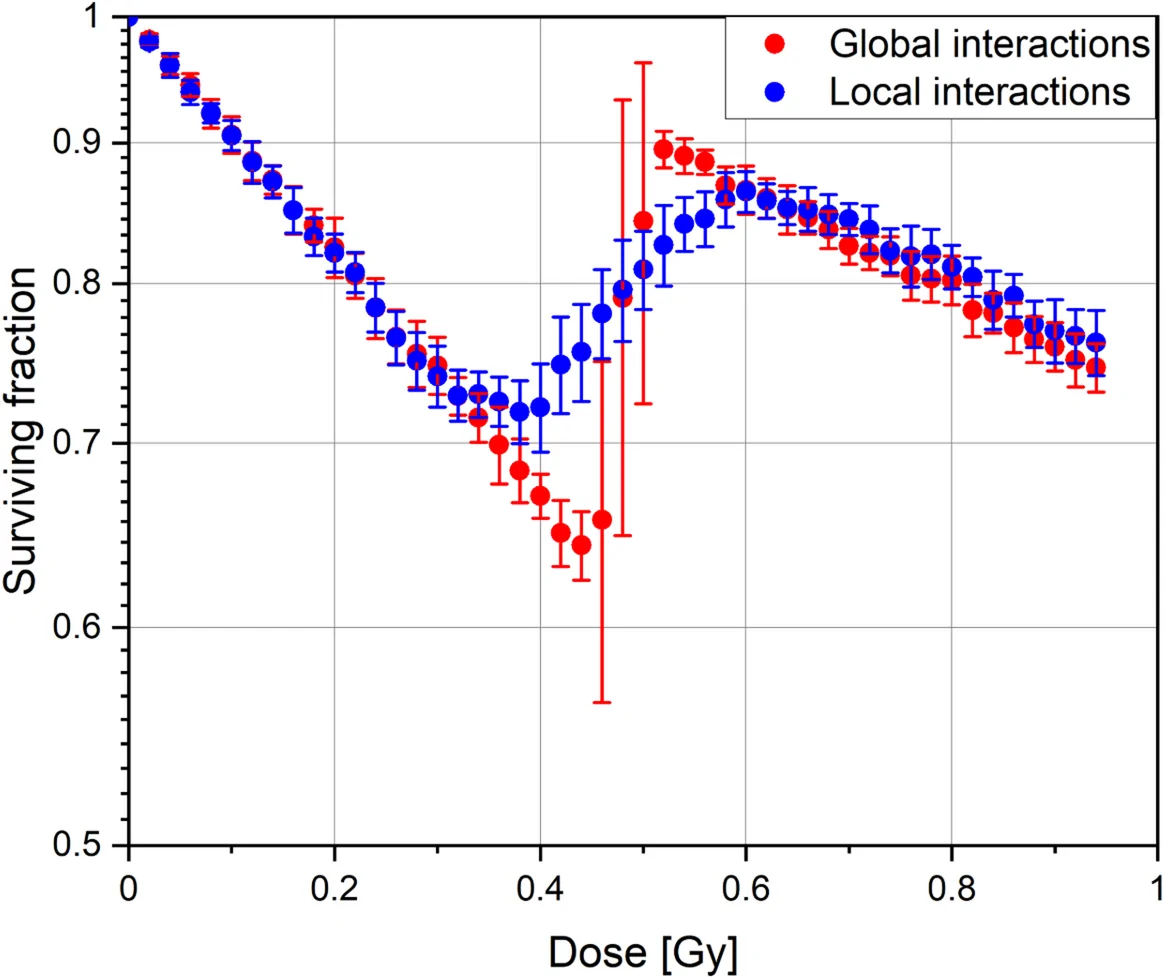
Comparison of global versus local decision-making in the MML model. When all cells respond to the global average damage (idealised global communication), survival increases sharply at a specific threshold dose, producing a near step-function in the survival curve. In contrast, when cells respond only to local neighbourhood damage (biologically plausible communication), the transition becomes gradual, resulting in the smooth shoulder region characteristic of IRR.

If the communication was global, this threshold would cause a single step of increase in cell survival. However, as the neighbouring average damage is location dependent, it smooths this single step into a shoulder region. This distinction between local and global interactions is illustrated in Fig. 10, which shows how spatially limited signalling produces a smooth shoulder in the survival curve, whereas global information leads to a sharp step-like increase in survival.

Other models propose an explanation in relation to the radiation induced bystander effect^34^. This refers to the phenomenon where non-irradiated, or non-targeted cells in the neighbourhood of an irradiated cell exhibit damage and show similar responses as the targeted cell, including changes in gene expression, cell proliferation, apoptosis, cell inactivation, or cell death^35–37^. Cell-to-cell communication is a necessary condition for the radiation-induced bystander effect^38^.

The biological implementation of the intercellular communication assumed in the MML model remains an important open question. In the present framework, signalling is represented in an abstract form: irradiated cells emit a damage-related signal, and neighbouring cells integrate this information when determining whether to survive or die to minimise the local mutation burden. This formulation is not intended to identify a specific molecular pathway, but rather to test whether local exchange of damage-related information is sufficient to reproduce HRS/IRR survival curves. Nevertheless, several known radiation-associated signalling mechanisms could contribute to such a process.

Secreted soluble factors, including cytokines, reactive oxygen species, and other signalling molecules, are plausible mediators of such paracrine communication. In addition, extracellular vesicles, including exosomes, are also relevant in this context because they can mediate cell–cell communication in the extracellular space. Their cargo can depend on the cell type and on the stress state of the donor cell, including exposure to ionising radiation^39^. Radiation exposure has also been reported to influence exosome release or uptake in a cell-type-dependent manner, suggesting that vesicle-mediated signalling could contribute to radiation-induced communication between cells^40,41^.

Gap junction communication represents a well-established pathway in radiation-induced bystander effects, but its relevance to the present model is limited. In clonogenic survival assays, cells are often sparsely distributed during irradiation and in the early post-irradiation period, limiting stable direct contacts between neighbouring cells and therefore reducing or eliminating gap-junction-mediated communication. For this reason, the present implementation of the MML model does not include gap junctions and instead uses a diffusible, distance-dependent signal as a simplified representation of paracrine communication.

The Minimum Mutation Load model presented in this paper also relies on damage recognition and communication between the cells, thus aligning conceptually with the bystander mechanisms. The MML model, however, also provides a potential explanation for why this communication exists. The reduction in the overall mutation burden in the tissue suggests an evolutionary advantage for maintaining these communication networks, as they may contribute to damage mitigation at the tissue level. In this sense, the communication term in the MML model should be interpreted as an effective representation of damage-related intercellular information, rather than as a direct description of a single signalling pathway.

The role of cell-to-cell communication in the model implies that surviving fractions depend on cell density. However, most published studies do not report the cell densities used in clonogenic assays, despite experimental evidence that cell density can significantly influence survival outcomes^42,43^. While parameters *σ*^2^, *R*, and *N* of the MML model can be adjusted to account for variations in cell density, constant values were used for all of them in order to avoid overparameterisation. The effect of cell density should be investigated in future studies using datasets in which plating density and culture conditions are explicitly reported. Future experimental work that manipulates communication pathways, such as soluble factor transfer, extracellular-vesicle release, or experimental conditions that promote direct cell–cell contact, could also help determine which mechanisms contribute most strongly to the response represented by the MML model.

Using the same number of parameters as the IR model for fitting to experimental data is also important to obtain a fair comparison with the mathematical model most frequently applied in relation to HRS and IRR. Our results show that the two models perform similarly in capturing the key features of the survival curves. However, unlike the IR model—which is entirely phenomenological and whose additional parameters (relative to the LQ model) cannot be independently determined—the MML model offers biologically meaningful parameters. The spontaneous mutation rate and the mutagenic damage induction rate in the MML model are defined independently of the clonogenic assay, and could, in principle, be measured through separate experiments.

While the values of the spontaneous mutation rate and the mutagenic damage induction rate obtained by the MML model vary across datasets, their magnitudes are broadly consistent with literature estimates. Considering an estimation for the range of somatic mutation rate between 0.3 × 10^− 9^ and 1.5 × 10^− 9^ mutation per base pair per cell division by Lynch^44^ and the size of the human genome of 6 × 10^9^ base pairs^45^, 2 to 9 mutations per cell division can be obtained, which are approximately one order of magnitude higher than that of the MML model. Albertini et al.^46^ reported ~ 7 × 10⁻⁶ HPRT mutations per cell per Gy; since the HPRT gene comprises about 1/140,000 of the genome, this corresponds to roughly ~ 1 mutation per cell per Gy across the whole genome. Given the simplicity of the model and its limited parameter set, this level of agreement with empirical estimates is encouraging.

Beyond comparison with literature estimates, the biological interpretation of the mutation-related parameters could be further tested experimentally. One strategy would be to irradiate cells, allow surviving cells to form progeny, and quantify mutation burden in the progeny using reporter mutation assays^47^, HPRT-type assays^48^, targeted sequencing^49^, whole-genome sequencing^50^, or single-cell sequencing^51^. The model predicts that conditions allowing intercellular communication are expected to reduce the mutation burden among surviving progeny compared with conditions in which communication is disrupted. This reduction may occur at the cost of increased low-dose cell elimination. Conversely, if disrupting intercellular communication changed neither the low-dose survival pattern nor the mutation burden of surviving progeny, this would argue against the proposed mechanism.

A central challenge in interpreting in vitro low-dose radiation responses is the difference between the experimental systems and the intact tissues featuring more complex biology. It is well understood that clonogenic survival assays—while widely used—cannot reproduce the full microenvironmental, architectural, and hierarchical context of multicellular organisms. These in vitro systems, nevertheless, remain essential tools in radiation biology, as they offer a high degree of experimental control and reproducibility, and they capture key aspects of cellular radiation response^5–7^, including DNA damage sensing, repair, and cell fate decisions.

In vitro systems may also retain functionally relevant aspects of mutation-load minimizing behaviour. In our interpretation, the mechanisms responsible for HRS and IRR —such as damage-dependent intercellular signalling and elimination of excessively damaged cells—may also be at least partially conserved in vitro. The observation that some cell lines display HRS and IRR while others do not may further support this interpretation, as it may reflect the heterogeneous retention or loss of such protective mechanisms during immortalization and passaging. While in vitro systems do not fully replicate in vivo biology, they provide a feasible platform for evaluating whether a principle such as mutation-load minimization through context-dependent cell death can reproduce known survival patterns across diverse experimental conditions. The success of the MML model in fitting a wide array of clonogenic datasets suggests that this hypothesis may capture one layer of the regulatory logic that may also function in tissues.

While the present study focused on in vitro systems, the MML framework can also be extended to real tissues by addressing three key aspects: geometry, signalling, and cellular hierarchy. In a three-dimensional system, each cell interacts with more neighbours at the same cell density, which stabilises the local average of the damage signal and reduces the extent of hypersensitivity in the survival curve. Regarding signalling, living tissues rely not only on secreted soluble factors but also on gap-junction–mediated communication, which provides additional information about the spatial distribution of mutagenic damage. As the fundamental concept of the model remains unchanged, similar HRS and IRR responses are expected, though with parameter values reflecting tissue-specific signalling characteristics. To capture the hierarchical organisation of tissues, mutagenic damages could be weighted according to the oncogenic potential of the affected cell type — for instance, assigning lower weights to mutations in terminally differentiated cells and higher ones to stem or progenitor cells.

While this study focused on radiation-induced hypersensitivity, the model should, in principle, be applicable to any form of mutagenic stress. Ionising radiation serves as a well-controlled experimental perturbation for inducing DNA damage, but the mutation minimisation effect is not dependent on the specific cause of damage. Rather, its underlying principle is to reduce genomic instability and, ultimately, cancer risk.

The MML model presented in this study supports a novel conceptual framework for understanding how multicellular tissues might actively manage mutation burden. Unlike traditional models that treat cell death as an intrinsic response, the MML approach assumes that survival decisions are modulated by local intercellular communication, enabling tissues to approximate an optimal trade-off between eliminating damaged cells and limiting the proliferation-associated risk of new mutations. This implies that tissues may operate as distributed systems, in which individual cells collectively minimise the total mutational load via simple local rules. While ionising radiation is used here as a well-controlled perturbation, the underlying principle—mutation minimisation through context-dependent cell fate decisions—could apply broadly to other sources of mutagenic stress or even to developmental quality control. Thus, the MML model offers a theoretical foundation for a new paradigm in tissue homeostasis: one in which somatic integrity is not merely a byproduct of cell-intrinsic repair and death pathways, but an emergent property of cooperative cellular behaviour shaped by evolutionary pressures to suppress cancer.

## Conclusions

This study introduced the Minimum Mutation Load model as a principle-based explanation for low-dose hyper-radiosensitivity and induced radioresistance. Grounded in the principle of minimising somatic mutation burden, the model assumes that cells make survival decisions based not only on their own damage but also on the local damage environment, mediated by intercellular signalling. Using a database of 99 clonogenic survival experiments, we showed that the MML model can reproduce key features of observed survival curves and performs comparably to the established Induced Repair model, despite having a clearer biological rationale and interpretable parameters. In contrast to the IR model, the MML model defines its parameters independently of clonogenic survival data, offering potential for broader biological interpretation and experimental validation. These results support the idea that tissues may act as cooperative systems to suppress genomic instability. While developed in the context of ionising radiation, the model’s underlying logic is more general, offering a potential framework for understanding tissue responses to various mutagenic stressors. Future studies may further test this hypothesis by exploring mutation burden and cell signalling experimentally.

## Data Availability

The experimental datasets analysed during the current study are available in the STORE^DB^ repository, https://doi.org/10.20348/STOREDB/1163/1252^14,15^.

## Code availability

The code used to implement the Minimum Mutation Load (MML) model and perform the simulations in this study is publicly available in a GitLab repository, https://gitlab.ek-cer.hu/FAIR/HRS-signalling. The code has also been archived in the generalist Zenodo repository, https://doi.org/10.5281/zenodo.20846430, and catalogued via the domain-specific STORE^DB^, https://doi.org/10.20348/STOREDB/1239/1362^52^.
